# von Willebrand disease combined with other hemostasis disorders: an overlooked clinical entity

**DOI:** 10.1016/j.rpth.2025.103259

**Published:** 2025-11-17

**Authors:** Omid Seidizadeh, Pier Mannuccio Mannucci, Flora Peyvandi

**Affiliations:** 1 Fondazione IRCCS Ca’Granda Ospedale Maggiore Policlinico, Angelo Bianchi Bonomi Hemophilia and Thrombosis Center and Fondazione Luigi Villa, Milan, Italy; 2 Department of Pathophysiology and Transplantation, Università degli Studi di Milano, Milan, Italy

**Keywords:** co-inheritance, combined bleeding disorders, diagnostic challenges, next generation sequencing, von Willebrand disease

## Abstract

von Willebrand disease (VWD) is common, affecting ∼1% of the general population. Due to this relatively high prevalence, it can co-occur with other coagulation disorders. However, combined defects are often underrecognized in clinical practice. Diagnostic anchoring, overlapping symptoms, and limited access to comprehensive hemostatic and genetic testing contribute to these missed diagnoses. A growing number of case reports, case series, and cohort studies have documented the coinheritance of VWD with hemophilia A or B, rare coagulation factor deficiencies, and platelet function disorders. In some cohorts, up to one-third of cases with mild VWD had an additional hemostatic defect. Emerging genomic data also suggest that multiple low-frequency variants affecting hemostasis can co-occur in the same individual, particularly in populations with founder effects or high rates of consanguinity. Combined defects can alter bleeding severity, complicate diagnosis, and affect treatment decisions. Broader awareness and multidisciplinary evaluation are critical for addressing this underappreciated aspect of inherited bleeding disorders.

## 1 Introduction

von Willebrand disease (VWD) is common, affecting ∼1% of the general population [1]. Although the prevalence of clinically significant cases is estimated to be lower (∼1 in 1000) [2], recent large-scale, multiancestry population-genomic studies suggest that the global prevalence may be underestimated [3,4]. Even assuming a 1% estimate, individuals with other bleeding disorders have a relatively high probability of also having VWD; meaning that in nearly every 100 cases of any bleeding disorder, 1 would also have VWD. This raises an important clinical concern: are patients with VWD being adequately tested for other coexisting bleeding disorders—and vice versa?

The first report of VWD co-occurring with another bleeding disorder (in this instance, hemophilia A) was published more than 4 decades ago [5]. Since then, several case reports, case series, and cohort studies have documented that combined VWD and other coagulation disorders represent a real scenario (Table) [[5], [6], [7], [8], [9], [10], [11], [12], [13], [14], [15], [16], [17], [18], [19], [20], [21], [22], [23], [24], [25]]. Despite growing recognition, current clinical practices less frequently account for combined hemostasis disorders.

**Table:** Reported cases and studies of von Willebrand disease combined with other coagulation disorders.

Study	Combined defects with VWD	Key findings/clinical notes
Pezeshkpoor et al. [6], 2025	Type 3 VWD and antithrombin deficiency	Family report; antithrombin deficiency reduced bleeding severity in type 3 VWD; relevant for personalized treatment.
Seidizadeh et al. [7], 2024	Low VWF and multiple other factor deficiencies	Frequency: 36% (89 of 250); mild/severe factor deficiencies and platelet disorders with reduced VWF.
Seidizadeh et al. [8], 2021	Various VWD types with multiple other factor deficiencies	Frequency: 19.2% (25 of 130); multiple coagulopathies with reduced VWF; important for treatment planning.
Preisler et al. [9], 2021	Various VWD types with multiple other factor deficiencies	Cohort study; next generation sequencing–based detection of combined deficiencies; 10 VWD cases of 52 with FV, FVII, FVIII, FIX, FXI, fibrinogen, or antithrombin deficiencies; important for treatment planning.
Pipe et al. [10], 2019	Type 2M VWD and FXIII deficiency	Case report; severe FXIII deficiency with reduced VWF; personalized management essential.
Lavin et al. [11], 2017	Low VWF and multiple other factor deficiencies	Frequency: 11% (10 of 91); mild factor deficiencies and platelet function disorders alongside reduced VWF.
Lavenu-Bombled et al. [12], 2016	Type 2B VWD and platelet-type VWD (PT-VWD)	Family report; thrombocytopenia, enhanced RIPA, reduced VWF and abnormal VWF multimer profile.
Robson and Mumford [13], 2009	VWD types with multiple other factor deficiencies	Data from the UK Haemophilia Centre Doctors Organisation; VWD combined with FXI, FIX, FV, and FVIII deficiencies
Siegmund et al. [14]	Type 1/2 VWD and hemophilia A/B	Frequency: 3.7% (5 of 134); severe FVIII or FIX deficiencies with reduced VWF; treatment adjusted in 2 cases.
O’Brien et al. [15], 2007	Type 1 VWD with hemophilia A/B or FXI deficiency	Frequency: 2.6% (6 of 227); mild/severe factor deficiencies with reduced VWF; important for counseling and treatment.
Asatiani and Kessler [16], 2007	Various VWD types with other factor deficiencies	16 cases described; mild to severe factor deficiencies with reduced VWF; relevance for genetic counseling.
Casonato et al. [17], 2001	Type 2N VWD and hemophilia A	Family report; low FVIII:C and VWF:FVIIIB/VWF:Ag ratio; importance for genetic counseling.
Bux-Gewehr et al. [18], 2000	Type 1/2 VWD and FXII deficiency	Frequency: 9.2% (25 of 270); mild FXII deficiency with reduced VWF.
Zeitler et al. [19], 1999	Type 1 VWD and FXII deficiency	25 cases with mild FXII deficiency and reduced VWF; fewer bleeding episodes than VWD alone.
Pernod et al. [20], 1996	Type 1 VWD and hemophilia B	Family report; mild hemophilia B and reduced VWF; significant for treatment planning.
Girolami et al. [21], 1993	Type 1 VWD and FVII Padua	Family report; heterozygous FVII Padua with reduced VWF.
Nounou and Spence [22], 1993	VWD and Glanzmann’s thrombasthenia	Family report; bleeding more severe with both conditions.
Tavori et al. [23], 1990	VWD and FXI deficiency	Frequency: 13% (54 of 400); mild FXI deficiency and reduced VWF; bleeders had lower VWF than nonbleeders.
Grand et al. [24], 1990	VWD and FXIII deficiency	Case report of severe FXIII deficiency with abnormal VWF levels.
Miller CH, et al. [25], 1986	Type 1 VWD and hemophilia A	5 families with combined deficiencies; important for carrier detection in hemophilia A.
Beddall et al. [5], 1984	Type 2A VWD and hemophilia A	Family report; severe hemophilia A with reduced VWF; treatment implications.

FVIII:C, Factor VIII coagulant activity; PT-VWD, platelet type von Willebrand disease; RIPA, ristocetin-induced platelet agglutination; VWD, von Willebrand disease; VWF, von Willebrand factor; VWF:Ag, von Willebrand factor antigen; VWF:FVIIIB, von Willebrand factor:factor VIII binding assay.

When co-occurring, overlapping features between VWD and other bleeding disorders can complicate diagnosis, delay targeted treatment, and obscure risk stratification for challenges such as surgery, menstruation, and childbirth. Combined defects are particularly likely to occur in disorders with a relatively high prevalence, such as VWD occurring alongside hemophilia A or B, factor (F)VII deficiency, FXI deficiency, and platelet function disorders. Nonetheless, case reports, case series, and cohort studies remain scarce in the literature (Table). The probability of coinheritance is expected to be even higher in populations where consanguinity is more common, further underscoring the need for comprehensive diagnostic approaches in these settings [8,22]. Additionally, in genetically isolated populations such as Ashkenazi Jews, founder mutations can lead to the increased prevalence of specific bleeding disorders such as FXI deficiency, further raising the likelihood of combined defects [23].

But why is the co-occurrence of VWD with other coagulation disorders often underrecognized? This is due to both diagnostic bias and practical constraints. Routine diagnostic workflows often follow a stepwise approach that stops once an abnormality is found. As a result, further testing, especially for rare factor deficiencies or platelet function defects, is frequently omitted. For example, in patients with FVII deficiency, a prolonged prothrombin time may draw disproportionate clinical attention, so that such other contributors to bleeding as, for instance, VWD may be overlooked. Conversely, VWD-related reductions of plasma FVIII can prolong the activated partial thromboplastin time, potentially masking concomitant deficiencies in intrinsic pathway factors like FVIII, FIX, FXI or FXII unless specifically investigated. In addition, a diagnosis of VWD may also deter clinicians from pursuing additional evaluations, reflecting a common tendency toward diagnostic anchoring, so that subsequent investigations are deprioritized once a plausible diagnostic explanation is identified. This is compounded by the phenotypic overlap between VWD and some other conditions like platelet disorders, particularly regarding such mucocutaneous symptoms as epistaxis, menorrhagia, or easy bruising, which may obscure the presence of a second underlying bleeding disorder. Moreover, although the 2021 VWD diagnostic guidelines acknowledge that concomitant bleeding disorders, such as platelet function defects, may contribute to bleeding, particularly in individuals with von Willebrand factor (VWF) levels between 30 and 50 IU/dL, current diagnostic algorithms still rarely offer explicit recommendations for systematically evaluating multiple concurrent defects, further contributing to their underrecognition [26]. The mild nature of concomitant defects and laboratory variability, such as VWF fluctuations with stress or hormones, can mask coexisting mild deficiencies.

The underutilization of next-generation sequencing and comprehensive phenotyping tools, especially in milder phenotypes, also plays a role because some patients may harbor multiple, coinherited defects with variable expressivity that elude conventional testing. Beyond underrecognition of the need for such testing, additional barriers such as limited access to specialized reference laboratories in certain geographic regions, high testing costs, and challenges with insurance authorization further restrict the use of these approaches. Therefore, expanded diagnostic evaluation should be considered when bleeding severity, pattern, or treatment response deviates from expectations, particularly in populations in whom consanguinity is common or genetic predisposition is likely to be higher.

Several studies, including few large cohorts, case series, and case reports, have documented coexisting hemostasis abnormalities in patients with VWD, and vice versa [[6], [7], [8], [9], [10], [11], [12], [13], [14], [15], [16], [17], [18], [19], [20], [21], [22], [23], [24], [25]]. These include VWD plus hemophilia A and B, rare coagulation defects (fibrinogen, FII, FV, FVII, FXI, FXII, and FXIII), platelet function disorders, antithrombin deficiency, lupus anticoagulants, Noonan syndrome, or vascular Ehlers-Danlos syndrome (summarized in the Table). In a cohort of 250 patients with low VWF/type 1 VWD, 36% (89 of 250) had an additional hemostasis defect [7]. The most common were platelet function disorders (22%), followed by deficiencies in FVII (19%), FXII (17%), and FXI (15%). Less frequent combinations included hemophilia A (6%), FV (3%), FXIII (2%), and FII (1%). Rare associations with reduced VWF included Noonan syndrome and vascular Ehlers-Danlos syndrome. Interestingly, 11 patients had more than 2 coexisting hemostatic abnormalities. The bleeding scores using the International Society on Thrombosis and Haemostasis-Bleeding Assessment Tool (ISTH-BAT) did not differ significantly between patients with combined defects and those with isolated reduced VWF, likely due to the influence of age, gender, and the mild phenotype of the secondary defects [7]. In addition, an Irish cohort study found the co-occurrence of low VWF/type 1 VWD and coagulation factor deficiencies in 11% of cases. Although the ISTH-BAT in cases with multiple defects was slightly higher than that in VWD only cases, the difference was not statistically significant [11]. An investigation of Iranian patients with VWD, mostly with a mild defect, also showed that ∼20% had a second or third hemostasis defect, with higher but not significantly different ISTH-BAT in cases with multiple defects [8]. A lower frequency of 3.7% has been documented for VWD combined with hemophilia A or B among 134 patients from Germany [14]. These patients exhibited severe deficiencies in FVIII or FIX (<1%) alongside reduced VWF, leading to changes in treatment regimens in some cases [14]. Similarly, a 2.6% frequency of type 1 VWD combined with hemophilia A, hemophilia B, or FXI deficiency was found among 227 patients from the United States, with relevance for both genetic counseling and treatment [15]. Notably, a family with severe type 3 VWD and antithrombin deficiency was recently reported, where the antithrombin deficiency significantly attenuating bleeding severity [6]. A 13% frequency of VWD combined with FXI deficiency among 400 patients was reported in Israel, with bleeders showing significantly lower VWF levels than nonbleeders [23]. Others described 25 cases, among 270 patients, of type 1 VWD combined with FXII deficiency (frequency of 9.2%), with these cases experiencing fewer bleeding episodes than those among patients with VWD alone [18]. At a national level, 2005 registry data from the United Kingdom Haemophilia Centre Doctors Organisation reported 21 cases of VWD combined with FXI deficiency, 3 with FIX deficiency, 2 with FV deficiency, and 2 with FVIII deficiency [13]. Case reports have described combined VWD and FXIII deficiencies, with more severe clinical phenotypes due to the coexistence of both defects [10,24].

The availability of large-scale genomic databases such as the Genome Aggregation Database has significantly advanced our understanding of the population frequency of disease-associated and benign variants. Notably, several *VWF* pathogenic variants have been identified at higher-than-expected frequencies, though still rare (minor allele frequency [MAF], <1%), suggesting that some individuals with other inherited bleeding disorders may also carry these variants, consistent with a concurrent diagnosis of VWD [3,4]. For example, the missense variants p.R852Q (associated with type 2N VWD; MAF, 0.5%) and p.Y1584C (type 1; MAF, 0.34%) and the gene conversions p.P1266L (type 2B; MAF, 0.067%), and p.P1266Q (type 2B or 2M; MAF, 0.054%) are all present at low but appreciable frequencies in multiancestry populations [3,4]. While these variants may not always manifest overt symptoms, they can contribute to clinically significant bleeding phenotypes, either independently in VWD cases or, more notably, when coinherited with other coagulation disorders, particularly during hemostatic challenges such as surgery or pregnancy.

Importantly, some patients are now recognized to harbor multilocus genetic defects that modify or obscure the classical phenotypic presentations of bleeding disorders. In a study of 52 patients with combined coagulation defects, VWD was frequently identified (*n* = 10) to occur alongside deficiencies in FV, FVII, FVIII, FIX, FXI, fibrinogen, and antithrombin [9]. Genetic analysis using next-generation sequencing revealed that most VWD cases (types 1, 2A, and 2M) co-occurred with other factor deficiencies due to independent heterozygous *VWF* missense variants. Only 1 case of type 3 VWD was identified, caused by a large homozygous deletion in the *VWF*. The use of gene panels and sequencing enhanced the detection of combined defects, helping to prevent underdiagnosis or misclassification [9].

Overall, these observations highlight the value of comprehensive and ethically guided genetic assessment in inherited bleeding disorders, particularly when the clinical phenotype is not fully explained by conventional testing. While next-generation sequencing has facilitated the identification of *VWF* variants and multilocus genetic defects, such testing should be performed within appropriate clinical or research frameworks and always with informed consent. Importantly, we do not advocate for indiscriminate or routine genomic testing, but rather its use when clinically indicated and ethically justified to help clarify complex or overlapping bleeding phenotypes. Interpretation of *VWF* variants can be challenging, as many remain classified as variants of uncertain significance, emphasizing the need for careful clinical correlation [[9], [27]].

Pertaining to the therapeutic approach to the combination of VWD with other inherited hemostasis disorders, the high phenotypic heterogeneity of the different combinations makes it difficult to provide univocal guidelines, so the approach must by definition be personalized case by case. For the index VWD cases, there are specific guidelines that should be tailored to the VWD subtype and bleeding tendency severity [28]. For the spectrum of combination defects, management depends on the severity of each defect and its impact on the clinical phenotype. For sake of examples, the combination of VWD with a platelet function disorder may demand the use of platelet transfusions that are not part of the established therapy of VWD. The combination of hemophilia A with VWD can simply be handled by using the replacement products that are used and efficacious for both diseases, such as FVIII/VWF concentrates and desmopressin. The relative frequent combination of VWD and FXI deficiency does not necessarily need to resort to FXI replacement therapy, and tranexamic acid may be sufficient to control bleeding, particularly for mucosal bleeds and mucosal tract surgeries. In combined coagulation defects such as FIX and FVII deficiencies, replacement of the deficient factor may be added to the treatment for VWD when the plasma levels are deemed insufficient for hemostasis.

## 2 Conclusions

Combination of VWD with other hemostasis disorders is often overlooked but may have important consequences not only for diagnosis and genetic counseling but also for treatment in some instances. A tendency to halt investigations after the first identified defect, together with limited access to comprehensive hemostatic and genetic testing, contributes to underrecognition. Emerging data from population-based studies and sequencing indicate that coinheritance of multiple bleeding-related variants is more common than previously appreciated. To improve patient care, clinicians and laboratory scientists should adopt a broader diagnostic perspective and consider additional phenotypic and, when appropriate, genetic evaluations in patients with unexplained or disproportionate bleeding or suboptimal treatment response, rather than focusing too narrowly on a single underlying cause.
